# The expression of kappa-opioid receptor promotes the migration of breast cancer cells in vitro

**DOI:** 10.1186/s12871-021-01429-z

**Published:** 2021-08-30

**Authors:** Huiqing Li, Zhenzhen Ma, Yunlong Lei

**Affiliations:** 1 Department of Anesthesiology, Shandong Provincial Third Hospital, No.11, Wuyingshan Middle Road, Tianqiao District, Jinan, 250031 Shandong China; 2 Department of Thoracic Surgery, Shandong ENT Hospital, Jinan, 250023 Shandong China

**Keywords:** OPRK1, Breast cancer, Opioid receptor, AKT, Migration

## Abstract

**Background:**

Opioid receptors are implicated in cell proliferation and cancer migration. However, the effects and underlying mechanisms of opioid receptor κ (OPRK1) in breast cancer remain unknown.

**Methods:**

Small interfering RNA (siRNAs) was used to knockdown the expression of OPRK1. Western blot was used to determine the protein expression and reverse transcription-quantitative PCR (RT-qPCR) determined the genes transcription. Cell viability was detected by MTT assay and cell death rates were determined by Annexin V/PI and flow cytometry. Cell migration and invasion were detected by wound healing analysis and transwell assay, respectively.

**Results:**

Our research demonstrated that OPRK1 was overexpressed in breast cancer cells compared with the normal human mammary epithelial cells. OPRK1 knockdown could inhibited cell viability and migration in cancer cells, accompanied with the decreased proteins and genes expression of N-cadherin, Snail, MMP2 and Vimentin, while the E-cadherin expression was increased. Additionally, OPRK1 knockdown also promoted PI3K/AKT signaling inactivation. Activation of AKT reversed the OPRK1 knockdown-induced cell viability inhibition and migration suppression, while inhibition of AKT reduced cell viability and promoted cell death.

**Conclusions:**

Our findings illustrated the role of OPRK1 played on promoting migration in vitro, and we also provided the therapeutic research of OPRK1 knockdown combined with AKT inhibition.

## Background

At present, postoperative recurrence and migration of malignant tumors are still difficult to control, which may be related to multiple factors affecting prognosis and their mechanism are still unknown, and anesthesia may be one of the influencing factors [1]. The influence of anesthetic drugs and methods on postoperative tumor growth and migration has attracted increasing clinical attention [2, 3]. Studies have shown that different anesthesia strategies have effects on tumor proliferation and migration, and there are also differences between different drugs and methods [4]. Opioid agonists, such as fentanyl, are powerful narcotic analgesics and are currently the first choice for clinical pain treatment, and it is found that opioids receptors might be involved in promoting cancer recurrence and migration [5, 6].

The opioid receptors, a subfamily of the family A G protein-coupled opioid receptor superfamily, consist of μ (OPRM1), δ (OPRD1), and κ (OPRK1), all of which activate inhibitory G proteins [7]. Retrospective analyses and experimental data suggest the effects of opioids on cancer progression, migration, and recurrence [8, 9]. There is evidence that opioids affect immune system function, angiogenesis, apoptosis, and invasion in a potentially deleterious manner [6]. OPRK1 expression has been reported to be associated with a significantly poorer prognosis and tumor migration in various cancers, such as esophageal squamous cell carcinoma (ESCC) [10], and liver metastases of small bowel and pancreas neuroendocrine tumors [11], and these results strongly suggest an essential role of OPRK1 in tumor growth and migration.

Breast cancer is a common type of malignant tumor in women, characterized by high morbidity and mortality. The increasing incidence of breast cancer in the world threat to women's health greatly. The general treatment for breast cancer includes surgical resection combined with chemotherapy and radiotherapy. However, with the high invasion and migration of breast cancer cells, it is necessary to explore the effects of anesthesia strategies during treatment of breast cancer. Previous studies have shown the impact of regional anesthesia on recurrence, migration, and immune response in breast cancer surgery [12–15], and some studies report that anesthesia drug promotes and increases cancer proliferation and migration via opioid receptors [16, 17]. Here, in this study, we aimed to research the effects of OPRK1 in migration in breast cancer. We compared the differences in expression of OPRK1 in normal cells and breast cancer cells, and determined the cell viability, migration after OPRK1 knockdown using small interfering RNA (siRNAs). Furthermore, Due to the essential effects of PI3K/AKT pathway in tumor migration [18, 19], we also investigated the correlation between OPRK1 and PI3K/AKT pathway, and detected how OPRK1 affected migration of breast cancer cells when AKT activation/inhibition.

## Methods

### Cell culture and reagents

MDA-MB-231, MDA-MB-435 and MCF-7 cells (human breast cancer cells), and MCF-10A cells (the normal human mammary epithelial cells) were purchased from the American Type Culture Collection (ATCC). The MDA-MB-231, MDA-MB-435 and MCF-7 cells were incubated in DMEM medium (Life Technologies, Grand Island, NY, USA) contained with 10% fetal bovine serum (FBS) and antibiotics including penicillin and streptomycin. MCF-10A cells were incubated in DMEM/F12 contained with 5% horse serum, insulin, EGF, cholera toxin and hydrocortisone. All cells were maintained at 37 °C with 5% CO_2_ in a humidified atmosphere. The cell lines were validated by short tandem repeat analysis prior to use, and in this study, mycoplasma infection was routinely detected. Recilisib and Buparlisib were purchased from MedChemExpress company (USA). Primary antibodies include OPRK1 (Abclonal Technology), E-cadherin (Abcam), N-cadherin (Abcam), MMP2 (Abcam), Snail (Abcam), Vimentin (Abcam) and GAPDH (Abclonal Technology).

### Western blot

RIPA buffer, and Bicinchoninic acid assay kit (Thermo Fisher Scientific, Inc.) were used to extract and quantify the total protein from cells. 8 ~ 12% SDS-PAGE separated the proteins for 60 min and transferred onto PVDF membranes (EMD Millipore). The membranes were blocked with 3% BSA for 1 h at room temperature, and then incubated at 4 °C for 8 h with primary antibodies. It was followed by IRDye800 conjugated secondary antibody for 1 h at 37 °C. Immunoreactive protein was detected with an Odyssey Scanning System (LI-COR Inc., Lincoln, Nebraska).

### Reverse transcription-quantitative PCR (RT-qPCR)

Total RNA was extracted, detected and reversed using TRIzol® reagent (Takara Bio, Inc.), a NanoDrop™ 2000 spectrophotometer (Thermo Fisher Scientific, Inc.), the HiScript II 1st Strand cDNA Synthesis kit (Vazyme Biotech Co., Ltd.) according to the manufacturer's protocol [20]. The following primers were used for qPCR:

GAPDH, Forward, 5′-ATTCCATGGCACCGTCAAGGCTGA-3′ and reverse: 5′-TTCTCCATGGTGGTGAAGACGCCA-3′;

N-cadherin, forward: 5′-TTTGATGGAGGTCTCCTAACACC-3′ and reverse: 5′-ACGTTTAACACGTTGGAAATGTG-3′;

E-cadherin, forward: 5′-CGAGAGCTACACGTTCACGG-3′ and reverse: 5′-GGGTGTCGAGGGAAAAATAGG-3′;

Snail, forward: 5′- CCAATCGGAAGCCTAACTACAG-3′ and reverse: 5′- GACAGAGTCCCAGATGAGCATT-3′;

Vimentin, forward: 5′- GAGAACTTTGCCGTTGAAGC-3′ and reverse: 5′- GCTTCCTGTAGGTGGCAATC-3′;

MMP2, forward: 5′- GTGCTGAAGGACACACTAAAGAAGA-3′ and reverse: 5′- TTGCCATCCTTCTCAAAGTTGTAGG-3′;

### siRNA transfections

Transfection of scramble control and *OPRK1* siRNA (50 nM) (synthesized by GenePharma) in cells were performed according to the manufacturer’s instructions of Lipofectamine 2000 (Invitrogen, Carlsbad, CA, USA) [21].

### Transwell assay

The transfected cells were collected, suspended in serum-free medium, then transferred to the upper lumen and precoated with matrix gel. Medium containing 10% FBS was added to the lower chamber. The cells remaining in the upper chamber were removed, and the cells passing through the membrane were fixed with paraformaldehyde and stained with 0.1% crystal violet. The staining cells were photographed and counted under an inverted microscope.

### Wound healing assay

Cells were plated into 6-well plates and cultured in DMEM with 10% FBS until they reached 70 ~ 80% confluence. The confluent cell monolayers were scratched using a 10 μL pipette tip and incubated in culture medium with 1% FBS. Images were captured using a LEICA DMi8 inverted microscope.

### MTT assay

The cells were digested and applied into a cell suspension. The cells were seeded into a 96 well plate with 5000 cells per well. After cell transfection, the cells were incubated for other 24 h of standard culture or treatment with agents, subsequently. Twenty microliter MTT reagent (5 mg/ml) was added to each well for cell incubation. 150 μL DMSO (Beyotime Biotechnology, Nanjing, China) then dissolved the purple formazan. A multifunctional plate reader (BD Biosciences) measured absorbance at a wavelength of 570 nm.

### Flow cytometry assay

Annexin-V/PI Apoptosis Detection kit (Beyotime Biotechnology) determine the apoptosis of cells. Cells were seeded into 6-well plates and received transfection, then harvested, and resuspended in 100 μL Binding Buffer. The cell suspension was stained with 5 μl Annexin V and 5 μL PI for 5 min. Cell apoptosis was detectedd via BD FACSCalibur flow cytometer. Data were analyzed using FlowJo software.

### Statistical analysis

Statistical analyses were performed using GraphPad Prism software (version 6.0; GraphPad Software Inc.). Comparisons among groups were analyzed using the unpaired Student's t-test or one-way ANOVA followed by Tukey's post hoc test. Data are presented as the mean ± SD from at least three independent experiments. *p < 0.05 and **p < 0.05 are considered to indicate a statistically significant difference.

## Results

### The expression of OPRK1 in breast cancer cells and normal human mammary epithelial cellsin vitro

**Fig. 1:**
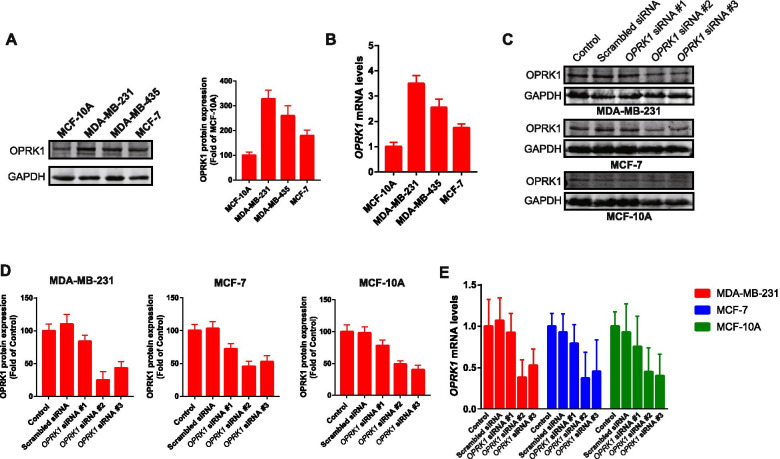
Expression of OPRK1 in breast cancer cells and normal human mammary epithelial cells. **A** OPRK1 protein expression was determined by western blot in MCF-10A, MDA-MB-213, MDA-MB-435 and MCF-7 cells. GAPDH was used as loading control. **B ***OPRK1* mRNA expression was determined by RT-qPCR in MCF-10A, MDA-MB-213, MDA-MB-435 and MCF-7 cells. **C-D** MDA-MB-231, MCF-7 and MCF-10A cells were transfected with *OPRK1* siRNA and the OPRK1 protein expression was determined by western blot. GAPDH was used as loading control. **E ***OPRK1* mRNA expression was determined by RT-qPCR in MDA-MB-231, MCF-7 and MCF-10A cells transfected with *OPRK1* siRNA

The cell lines of breast cancer cells including MDA-MB-231, MDA-MB-435 and MCF-7 cells as well as normal human mammary epithelial cells of MCF-10A was used to determine the protein expression of OPRK1 by western blot and RT-qPCR. As shown in Fig. 1A, the expressions of OPRK1 were different among the cell lines above. After qualification of proteins expression, the OPRK1 expressions were higher in breast cancer cells than normal cells. This result was also proved by RT-qPCR assay (Fig. 1B). Here, the breast cancer cell lines of MDA-MB-231 and MCF-7 were chosen to detected the function of OPRK1 in migration of breast cancer, as MDA-MB-231 cells with high expression of OPRK1 while the expression of OPRK1 in MCF-7 was low. In addition, the normal cell of MCF-10A was used as comparison. Previous studies showed that the migration ability was stronger in MDA-MB-231 cells than MCF-7 cells [22], and our results suggested the overexpression of OPRK1 might be associated with the migration ability of breast cancer cells. Therefore, we used siRNA to knockout the expression of OPRK1 and the cells of MDA-MB-221, MCF-7 and MCF-10A were transfected with three kinds of siRNA. Our results of western blot (Fig. 1C-D) and RT-qPCR (Fig. 1E) indicated that #2 siRNA had the highest efficiency on OPRK1 knockdown, and we used it for further research. The results suggested that OPRK1 was highly expressed in breast cancer cells both in translation and transcription, compared with the normal cells. Besides, MDA-MB-231 cells with high expression of OPRK1 and MCF-7 cells with low expression were used in subsequent studies, researching the changes of migration and differences between the two cells after OPRK1 knockdown.

### OPRK1 expression promoted cell viability and cell migration in breast cancer cells

**Fig. 2:**
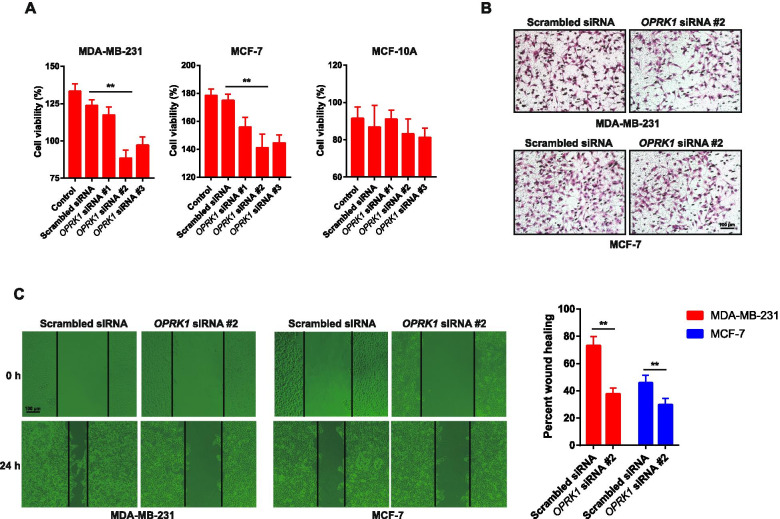
Role of OPRK1 in breast cancer cells and normal human mammary epithelial cells on proliferation, migration and invasion. **A** The cell viability was determined by MTT assay in MDA-MB-231, MCF-7 and MCF-10A cells transfected with *OPRK1* siRNA. **B** Invasion of MDA-MB-231 and MCF-7 cells transfected with *OPRK1* siRNA were subjected to transwell assay. **C** Migration of MDA-MB-231 and MCF-7 cells transfected with *OPRK1* siRNA were subjected to wound healing analysis, representative images (left) and statistical analysis (right) are shown

Migration is the risk factor that contributes to the high mortality rate in breast cancer [23]. In order to determine the effects of OPRK1 on migration of breast cancer cells, the *OPRK1* siRNA was used to knockdown the protein expression and compared the changes before transfection. Firstly, the cell viability was determined by MTT assay. After transfected with three kinds of *OPRK1* siRNA in MDA-MB-231 and MCF-7 cells, the cell viability decreased. And the #2 *OPRK1* siRNA had the most obvious reduction on cell viability both in MDA-MB-231 and MCF-7 cells compared with the cells transfected with scrambled siRNA (Fig. 2A), as #2 *OPRK1* siRNA had the most obvious reduction on OPRK1 expression (Fig. 1C-E). On the other hand, #1 *OPRK1* siRNA had the less effects on OPRK1 knockdown, and its cell viability inhibition effects was weaker than #2 *OPRK1* siRNA (Fig. 2A). It is suggested that OPRK1 expression promoted cell viability in breast cancer cells. However, in normal human mammary epithelial cells MCF-10A, the cell viability was not influenced by *OPRK1* siRNA (Fig. 2A), indicating that the effect of OPRK1 on cell viability might be different between normal cells and tumor cells. And then, we determined whether OPRK1 knockdown affect the migration in breast cancer cells. The transwell assay was used to detected the invasion ability of cancer cells. After transfected with #2 *OPRK1* siRNA in MDA-MB-231 and MCF-7 cells, we found that siRNA knockdown of OPRK1 significantly decreased their invasion ability compared with scrambled group (Fig. 2B). We also used a wound healing assay to evaluate the effect of OPRK1 knockdown on the migration ability of MDA-MB-213 and MCF-7 cells. The results showed that cell migration ability was significantly decreased in the OPRK1 knockdown group compared with scrambled group (Fig. 2C). However, we also found that in MCF-7 cells, with the lower migration ability than MDA-MB-231 cells [24], the cell migration ability change was also less sensitive to *OPRK1* siRNA (Fig. 2C). Therefore, knockdown of OPRK1 inhibited the invasion and migration of breast cancer cells in vitro.

### The effects of OPRK1 knockdown on the expression of migration-associated factors in breast cancer

**Fig. 3:**
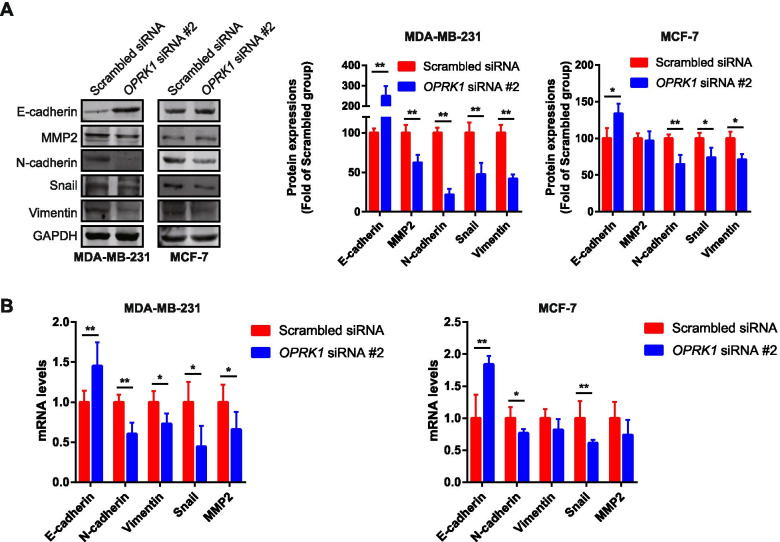
The effects of OPRK1 on the migration-related proteins and genes expression in breast cancer cells. **A** The protein expressions of E-cadherin, MMP2, N-cadherin, Snail and Vimentin were determined by western blot in MDA-MB-231 and MCF-7 cells transfected with *OPRK1* siRNA. GAPDH was used as loading control. **B** The genes expression of *E-cadherin*, *MMP2*, *N-cadherin*, *Snail* and *Vimentin* were determined by RT-qPCR in MDA-MB-231 and MCF-7 cells transfected with *OPRK1* siRNA

Epithelial-mesenchymal transition (EMT) has been shown to play a crucial role in promoting migration and invasion of cancer cells, and the marker of EMT including E-cadherin, N-cadherin, Snail [25]. We also determined the expression of Vimentin and matrix metalloproteinases 2 (MMP2). In MDA-MB-231 and MCF-7 cells, after treatment with #2 *OPRK1* siRNA, the western blot results showed that protein expression of epithelial maker E-cadherin was increased, while the expression of mesenchymal markers N-cadherin was decreased, as well as the expressions of Snail, MMP2 and Vimentin were also decreased (Fig. 3A). After protein quantification, we found that the protein expression changes were more significant in MDA-MB-231 cells than MCF-7 cells (Fig. 3A). It might be owing to the higher expression of OPRK1 in MDA-MB-231 cells. On the other hand, the mRNA expression also indicated the same results. The MDA-MB-231 and MCF-7 cells transfected with #2 *OPRK1* siRNA showed lower expression of N-cadherin, Vimentin, MMP2 and snail mRNA, while the E-cadherin mRNA expression was higher (Fig. 3B). The mRNA expression change was also more notable in MDA-MB-231 cells. These results verified that OPRK1 promoted cell migration in breast cancer cells in vitro.

### PI3K/AKT pathway activation inhibited the OPRK1 knockdown-decreased cell viability in breast cancer

**Fig. 4:**
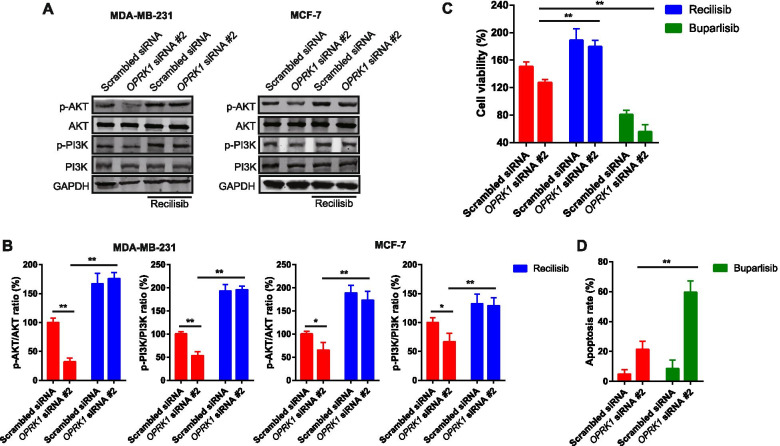
The effects of PI3K/AKT signaling on cell survival of breast cancer cells with OPRK1 knockdown. **A-B** MDA-MB-231 and MCF-7 cells transfected with *OPRK1* siRNA were treated with Recilisib for 24 h, and the protein expressions of p-AKT, AKT, p-PI3K, PI3K were determined by western blot. GAPDH was used as loading control. **C** MDA-MB-231 cells transfected with *OPRK1* siRNA were treated with Recilisib or Buparlisib for 24 h, and the cell viability was determined by MTT assay. **D** MDA-MB-231 cells transfected with *OPRK1* siRNA were treated with Buparlisib for 24 h, and the cell death rates were determined by Annexin V/PI staining and flow cytometry

Previous studies indicated that the PI3K/AKT pathway not only promotes cell survival and proliferation, but also controls EMT and cell migration in breast cancer [26, 27]. Here, we investigated the role of PI3K/AKT activation played in breast cancer cell after transfected with *OPRK1* siRNA. Firstly, the expression of PI3K and AKT were measured in MDA-MB-231 and MCF-7 cells, and we found that *OPRK1* siRNA transfection decreased the activation of AKT and PI3K, as the p-AKT and p-PI3K expression decreased, and the total protein expression of AKT and PI3K were stable (Fig. 4A). After protein qualification, the results showed that the reduction of p-AKT/AKT and p-PI3K/PI3K ratio was more significant in MDA-MB-231 cells than MCF-7 cells (Fig. 4B). It is suggested that the AKT and PI3K activation could be affected by OPRK1 expression in breast cancer cells, and the activation changed more notable in the cells with high migration ability. Therefore, we chose MDA-MB-231 cells for further research. Here, we also used the Recilisib, a compound that could activate PI3K/AKT signaling pathway, to determine its effects on the PI3K/AKT pathway activation. Our results showed that Recilisib promoted p-AKT and p-PI3K expression, indicating the PI3K/AKT activation was promoted by Recilisib. Recilisib also promoted PI3K/AKT activation in the cells transfected with *OPRK1* siRNA (Fig. 4A). And then, we determined the cell viability in MDA-MB-231 cells transfected *OPRK1* siRNA. The results showed that Recilisib reversed the cell viability inhibition induced by *OPRK1* siRNA significantly (Fig. 4C). Thus, we also used Buparlisib, a PI3K inhibitor. After treatment with Buparlisib, *OPRK1* siRNA-induced cell viability was promoted notably in MDA-MB-231 cells (Fig. 4C). In addition, due to the cell survival regulation of PI3K/AKT pathway, we determined whether cell death was triggered by combination of *OPRK1* siRNA and Buparlisib. And the results showed that Buparlisib promoted apoptosis of MDA-MB-231 cells with *OPRK1* siRNA transfection (Fig. 4D). The cell death induction effects of combination of *OPRK1* siRNA and Buparlisib might resulted from PI3K/AKT pathway inhibition, which promoted cell death signaling activation. These results suggesting that PI3K/AKT pathway activation reversed the cell viability inhibition induced by OPRK1 knockdown in vitro.

### PI3K/AKT pathway activation inhibited the OPRK1 knockdown-decreased cell migration in breast cancer

**Fig. 5:**
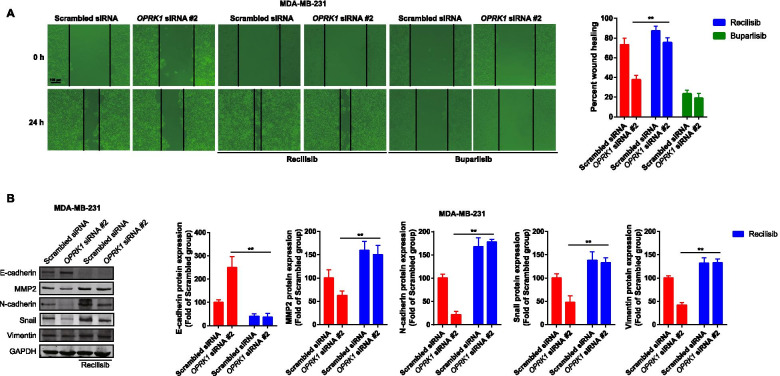
The effects of PI3K/AKT signaling on migration and related protein expressions of breast cancer cells with OPRK1 knockdown. **A** MDA-MB-231 cells transfected with *OPRK1* siRNA were treated with Recilisib or Buparlisib for 24 h, and the migration was performed by wound healing analysis, representative images (left) and statistical analysis (right) are shown. **B** MDA-MB-231 cells transfected with *OPRK1* siRNA were treated with Recilisib or Buparlisib for 24 h, and the protein expressions of E-cadherin, MMP2, N-cadherin, Snail and Vimentin were determined by western blot. GAPDH was used as loading control

We had proved that Recilisib reversed PI3K/AKT signaling pathway inhibition and cell viability inhibition induced by OPRK1 knockdown in MDA-MB-231 cells. And then, we determined whether PI3K/AKT re-activation could reverse OPRK1 knockdown-induced cell migration inhibition. The cell migration was determined by wound healing assay. The results showed that Recilisib promoted migration in MDA-MB-231 cells treated alone (Fig. 5A). Recilisib also promoted cell migration in the cells transfected with *OPRK1* siRNA. However, the PI3K inhibitor Buparlisib could further inhibited the cell migration in the cells transfected with *OPRK1* siRNA (Fig. 5A). It is suggested that PI3K/AKT signaling pathway was involved in the regulation on migration by OPRK1 expression. In addition, we also determined the expression of migration-associated factors. The western blot results showed that Recilisib could reversed the effects of *OPRK1* siRNA on proteins expression in MDA-MB-231. Recilisib treatment decreased E-cadherin protein expression, and promoted the protein expression of MMP2, N-cadherin, Vimentin and snail (Fig. 5B). It is also suggested the role of PI3K/AKT signaling played on OPRK1 expression-mediated migration in vitro*.*

## Discussion

The potential impact of surgery and anesthesia on cancer recurrence was reviewed to provide guidance for cancer surgical treatment [28]. Opioids remain the mainstay for treating cancer patients with pain management [29]. And OPRK1 expression shows the correlation with tumor progression in various cancers [10, 11]. Here, we verified the OPRK1 expression was enhanced significantly in breast cancer cells compared with normal cells. After knockdown of OPRK1, the cell viability and tumor migration were decreased notably. It is indicated that OPRK1 promoted cell migration in breast cancer, suggesting a therapeutic target for breast cancer patients.

In this study, we aimed to investigated the anti-migration of OPRK1 knockdown in normal cells MCF-10A and breast cancer cell lines. Interestingly, the cell viability was stable in MCF-10A cells after OPRK1 down-regulation, whereas it was inhibited in cancer cells. The selective inhibition on cell viability of OPRK1 knockdown suggested the correlation between OPRK1 expression and tumor proliferation. In addition, we chose the cell lines of MCF7 and MDA-MB-231 for further research due to the diversity of OPRK1 expression. Previous studies showed the differences in a comparative approach to weakly metastatic MCF-7 and strongly metastatic MDA-MB-231 breast cancer cell lines [24]. We also found that OPRK1 knockdown played the different effects on cell viability and migration in MCF-7 and MDA-MB-231. In our experiments, #2 *OPRK1* siRNA had the similar knockdown efficiency on *OPRK1* mRNA transcription in MCF-7 and MDA-MB-231 (Fig. 2E). However, the migration inhibition was more significant in MDA-MB-231 cells than MCF-7 cells (Fig. 2C). Furthermore, the migration-related proteins and genes expression also revealed that MDA-MB-231 cells with high migration ability were more sensitive to OPRK1 knockdown (Fig. 3A-B). It is proved the correlation between migration and OPRK1 expression, and suggested that OPRK1 regulation might be more efficiency in the cells with high migration.

Many works revealed the AKT activation promotes cell survival and plays the protective role against cell death during OPRK1 stimulation [30–33]. Besides, the activated AKT kinase is necessary for many events of the metastatic pathway including escape of cells from the tumor's environment, into and then out of the circulation, activation of proliferation, blockage of apoptosis [34, 35]. Therefore, the role of AKT activation or inhibition was initially researched in our study. Our results showed that AKT activation could reverse the migration inhibition induced by OPRK1 knockdown, including the expression inhibition of migration-related proteins (Fig. 5A-B). On the other hand, the AKT inhibition promoted the cell viability inhibition and cell death in the cells transfected with *OPRK1* siRNA (Fig. 4C-D). Furthermore, OPRK1 knockdown reduced PI3K and AKT activation and it was more significant in MDA-MB-231 cells than MCF-7 cells (Fig. 4B). It illustrated that the migration inhibition induced by OPRK1 knockdown might via PI3K/AKT suppression. Therefore, the OPRK1 suppression combined with AKT inhibition might be a strategy to against tumor growth, proliferation and migration.

This study also might propose the relationship between the roles of OPRK1 on tumor progression and the impact of anesthesia or analgesia management on cancer prognosis. Although OPRK1 expression has been reported to be associated with a significantly poorer prognosis and tumor migration in various cancers, such as esophageal squamous cell carcinoma (ESCC) [10], and liver metastases of small bowel and pancreas neuroendocrine tumors [11], the downregulation of OPRK1 in hepatocellular carcinoma (HCC) tumor tissues has a strong association with poor prognosis and OPRK1 might be a potential tumor suppressor [36]. Similarly, morphine, an agonist of the μ and k receptors [37], was reported that promotes and increases cancer proliferation and migration, while in other studies showed it prevents cancer progression. It is postulated that opioid receptors might play the opposite effect on different cancer cell types [17]. Therefore, Therefore, the role of opioid receptors in tumors needs to be studied according to the type of tumor. And our results showed that OPRK1 expression was higher than normal human mammary epithelial cells and was associated with tumor proliferation and migration. Our results were also proved by a result, which illustrated Naloxone, an opioid antagonist acting at the level of opioid receptors (μ, δ, and κ), can reduce breast cancer progression [38].

## Conclusions

In conclusion, our findings illustrated the role of OPRK1 played on promoting migration, and it was overexpression in breast cancer cells in vitro. This study might propose the relationship between the roles of OPRK1 on tumor progression and the impact of anesthesia or analgesia management on cancer prognosis. And we provided the therapeutic research of OPRK1 knockdown combined with AKT inhibition.

## Data Availability

The datasets used during the present study are available from the corresponding author upon reasonable request.

## Abbreviations

OPRK1: Opioid receptor κ
siRNAs: Small interfering RNA
RT-qPCR: Reverse transcription-quantitative PCR
ESCC: Esophageal squamous cell carcinoma
ATCC: American Type Culture Collection
FBS: Fetal bovine serum
EMT: Epithelial-mesenchymal transition
MMP2: Matrix metalloproteinases 2
HCC: Hepatocellular carcinoma
